# The role of minimally invasive radical hysterectomy for cervical cancer: ESGE-SERGS position document and joint-statement *

**Published:** 2020-05-07

**Authors:** Working Group of ESGE and SERGS, T Ind, L Mereu, R Verheijen, R Rovira Negre, V Zanagnolo, H Nassir, R Kimmig, G Scambia

**Affiliations:** Department of Gynaecological Oncology Royal Marsden Hospital London, United Kingdom;; St George’s University of London, United Kingdom;; Department of Obstetrics and Gynaecological S. Chiara Hospital Trento, Italy;; Department of Gynaecological Oncology, University Medical Center Utrecht, Netherlands;; Department of Obstetrics and Gynecology Hospital de la Santa Creu i Sant Pau Barcelona, Spain;; Department of Gynecologic Oncology Istituto Europeo Oncologico Milano, Italy;; Department of Obstetrics and Gynecology Poissy University Hospital Poissy, France;; Department of Obstetrics and Gynecology University Hospital Duisburg Essen, Germany;; Dipartimento per la Tutela della Salute della Donna e della Vita Nascente, Fondazione Policlinico Gemelli IRCCS;; Gynaecologic Oncology, Catholic University of Sacred Heart Rome, Italy.

**Keywords:** radical hysterectomy, minimally invasive surgery, cervical cancer, statement

## Abstract

Over the last two decades, minimal access techniques have gained widespread acceptance as an approach to radical hysterectomy for cervical cancer.

Two recent studies, the randomised study by Ramirez et al. (2018) and the epidemiologic study by Melamed et al. (2018) found that minimally invasive surgery radical hysterectomy for cervical cancer was associated with shorter overall survival than open surgery.

In this document we assess the importance of these two new studies and what their additional contribution is towards existing studies into the surgical approach to cervical cancer. Furthermore, we provide a consensus statement of the European Society Gynaecological Endoscopy (ESGE) and the Society of European Robotic Gynaecological Surgery (SERGS) as to the position of minimal access techniques (both standard and robotic) in light of this new evidence.

## Background

### 

Over the last two decades, minimal access techniques have gained widespread acceptance as an approach to radical hysterectomy for cervical cancer.

The evidence advocating laparoscopic over open approaches for cervical cancer has been based on a number of retrospective studies that have been summarised in two meta-analyses (Shazly et al., 2015; Wang et al., 2015). These studies found that a minimal access approach to cervical cancer surgery was associated with less blood loss, less post-operative complications, and a shorter hospital stay compared with open surgery with no difference in survival (Shazly et al., 2015; Wang et al., 2015). Subsequent studies with a combined cohort of over 1000 patients have shown similar findings (Sert et al., 2016; Shah et al., 2017; Corrado et al., 2018).

In 2018, the publication of two studies has resulted in the efficacy of a minimal access approach for cervical cancer to be questioned (Melamed et al., 2018; Ramirez et al., 2018). The first is an analysis of data from the USA National Cancer Database (NCDB) (Melamed et al., 2018). That study reports both a lower survival in women who have had a minimally invasive radical hysterectomy compared to open surgery and also demonstrated that the adoption of laparoscopic surgery coincided with a lowering of the four-year relative survival rate (Melamed et al., 2018). The second is a randomized controlled study (LACC trial) comparing laparoscopic and open radical hysterectomy for cervical cancer and reported that minimally invasive radical hysterectomy was associated with lower rates of disease free and overall survival compared to an open approach (Ramirez et al., 2018). Moreover, data on adverse events from the LACC trial have been recently published (Obermair et al., 2019) without finding any difference in terms of intra-operative and post-operative complications between minimal invasive and open surgery. This is in contrast with several meta-analyses based on non-randomised studies on cervical cancer and with multiple randomised controlled trials (RCT) and a Cochrane meta-analysis comparing open and laparoscopic hysterectomy in endometrial cancer (Galaal et al., 2018).

The National Clinical Cancer Network (NCCN) guidelines of 2019 state that ‘Previous iterations of the guidelines indicated that radical hysterectomy could be performed via open laparotomy or minimally invasive surgery (MIS) laparoscopic approaches, using either conventional or robotic techniques’. The guidelines then state, ‘Given recently presented findings of significantly poorer survival outcomes with the minimally invasive approach compared to the open approach in a randomized controlled trial of women with early-stage cervical cancer, women should be carefully counselled about the short-term versus long-term outcomes and oncological risks of the different surgical approaches’ (National Clinical Cancer Network; 2019). Despite the European Society of Gynaecological Oncology(ESGO)/ European Society for Radiotherapy and Oncology (ESTRO)/European Society of Pathology (ESP) guidelines of 2018 stating that a ‘minimally invasive approach is favored’ (Cibula et al.,2018.) a recent survey among ESGO members showed that 57% of responders had already changed their approach to open surgery a few months after the LACC trial results. Moreover 50% of members, still consider MIS to be appropriate for small tumors.

In this document we assess the importance of these two new papers and what their additional contribution is towards existing studies into the surgical approach to cervical cancer. Furthermore, we provide a consensus statement of the European Society Gynaecological Endoscopy (ESGE) and the Society of European Robotic Gynaecological Surgery (SERGS) as to the position of minimal access techniques (both standard and robotic) in light of this new evidence.

### A Cohort Study (Melamed et al., 2018)

This study reported on a cohort study looking at women who underwent radical hysterectomy for stage IA2 and IB1 cervical cancer and included 2461 women in the combined arms of both minimal access (n = 1225) and open approaches (n = 1340). Data were obtained from the NCDB and covered the years 2010 to 2013. There was a second part to this paper which was an interrupted time series analysis involving women who underwent radical hysterectomy for cervical cancer during the period of 2000–2010 using data from the Surveillance, Epidemiology, and End Results (SEER) database. For the NCDB analysis, the authors reported that over a median follow-up of 45 months, the 4-year mortality from any cause was 9.1% among women who underwent minimally invasive surgery and 5.3% among those who underwent open surgery (hazard ratio, 1.65; 95% CI, 1.22 to 2.22; P=0.002). One patient who had minimal access surgery died peri- operatively compared to three in the open group.

Even if the apparent quality of this study comes from the large number of patients included, another meta-analysis (Wang et al., 2015) that reported on survival differences between open and laparoscopic surgery and an additional two papers published subsequently (Sert et al., 2016, Corrado et al., 2018) with similar number of patients totalling 2334, show no differences in the recurrence rate.

Moreover, some comments can be made in respect to the multivariate analysis that has been reported in more detail in the supplement to the paper. Variables in the Cox multi-variate analysis included age, race, insurance type, grade, lymph node status, tumour size and adjuvant treatment. The authors justified their choice of variables in the supplement. However, histological type was not included in the multi-variant analysis even though there were significantly more cases of adenocarcinoma in the laparoscopic arm. Instead, histology comparisons have been shown as a separate uni-variant sub- analysis. Furthermore, the incidence of parametrial invasion and positive margins were not included in the Cox proportional hazard model and although not significantly so, there were more of those in the laparoscopic arm compared to open surgery.

The analysis of the SEERS database covered a different time period (2000-2010) to that of the NCDB. The total numbers could be calculated from the supplement to the paper and consist of about 437/ 5991 (7.3%) of women who received a minimal access approach. The article reported that before the adoption of minimally invasive radical hysterectomy (2000–2006), the 4-year relative survival rate among women who underwent radical hysterectomy for cervical cancer remained stable and that the adoption of minimally invasive surgery coincided with a decline in the 4-year relative survival rate of 0.8% (95% CI, 0.3 to 1.4) per year after 2006 (P=0.01 for change of trend). The authors have used a calculated temporal trend analysis based on a statistically non-significant drift observed prior to the introduction of MIS. Furthermore, the use of ‘relative’ survival over actual cancer-specific survival has resulted in improved results for the pre-2006 era. The authors have included the actual figures obtained from the Surveillance Epidemiology and End Result (SEER) database in their supplement and the four-year cancer-related survival both before and after January 2006 is almost identical in both arms at about 92%.

Another consideration about this article is the fact that it covered a period when MIS has been performed by early adopters both for standard laparoscopy and robotics. Furthermore, for robotics, the dates covered a time prior to the introduction of third and fourth generation robotic technology. This is important to recognise since in this study nearly 80% of the MIS cases were performed using a DaVinci robot.

The main critics of ‘big data’ studies consistently point to the population being examined and the accuracy of the data entered. Whether it should bear more weight over other observational studies is controversial. The data from the NCDB cohort is already out of date and we eagerly look forward to the data from 2014 to 2016. The SEER data stopped at 2010 and could have been extended to 2013 also.

### A Randomised Controlled Study – Laparoscopic Approach to Cancer of the Cervix (LACC) (Ramirez et al., 2018)

The study reported a phase 3 RCT comparing 312 open radical hysterectomies with 319 laparoscopic or robotic radical hysterectomies in women with early- stage cervical cancer. The data and safety monitoring committee called for an early closure of the trial. This interim analysis, on almost 81% of patients accrued, revealed that the disease-free survival rate at 4.5 years was lower with MIS compared to open surgery (86.0% vs. 96.5%; difference -10.6%, 95%CI -16.4 to -4.7%) and that MIS had a lower 3-year rate of overall survival (93.8% vs. 99.0%; hazard ratio for death, 6.00; 95%CI 1.77 to 20.30).

Designing a surgical RCT is notoriously difficult as it is impossible to control for different surgeons’ skills for different procedures. To ensure an adequate quality of MIS surgery, participating sites required accreditation from the trial management committee that involved a submission of peri-operative outcomes from a minimum of any ten MIS radical hysterectomies and two unedited videos of a MIS Piver-Rutledge type III radical hysterectomy.

It is unknown what the criteria was for assessing the videos and how many potential surgeons were rejected (Fader et al., 2018; Kimming et al., 2018). This resulted in 297 women actually receiving a MIS approach among 33 centres. The fact that all the recurrences were clustered in 14 of 33 participating centres raises questions as to whether there were unique surgical factors affecting the result or perhaps these centres were bigger recruiters or started recruiting earlier.

It is not that the minimally invasive arm performed so much worse in the LACC study, but that open surgery performed unexpectedly well. There were only seven recurrences in 312 (2.2%) women in the open surgery arm. This is an extremely low percentage of recurrence. On the other hand, 27 (8.4%) recurrences in the minimally invasive surgery (MIS) arm corresponded well to the data reported in most large studies comparing robotic surgery with open surgery for cervical cancer (Sert et al., 2016; Shah et al., 2017; Ramirez et al., 2018; Wang et al., 2015a,b; Derks et al., 2018; Gortchev et al., 2012; Zanagnolo et al., 2016). These studies have had recurrence rates for abdominal radical hysterectomy varying between 6,6 %-20% respectively (Sert et al., 2016; Shah et al., 2017; Ramirez et al.,2018; Wang et al., 2015a,b; Derks et al., 2018; Gortchev et al., 2012; Zanagnolo et al., 2016).

The difference between MIS and open surgery for the 3-year overall survival (93.8% vs 99%) was wider compared to other studies present in the literature including the Melamed study (Melamed et al., 2018) where a 4-years OS for MIS was 90.9% compared to 94.7% for open surgery. The sample size calculation of the LACC study itself was based on a disease- free survival rate of 90% in the open surgery arm. In the LACC study authors explained in the discussion that such difference is due to different analysis conducted: sequential comparison for retrospective study and concurrent analyses for RCTs.

One criticism of the study is the lack of a central histology review. In table S1 of the paper relative to postoperative histological characteristics there is a certain amount of “not reported data” on grade, invasion, vaginal resection margins, lympho-vascular space invasion (LVSI), and parametrial involvement that do not match with the amount of cases reported with “no residual disease”. These parameters have tremendous influence on recurrence-free survival (Derks et al., 2018) and the absence of a central histological review is a lesson for future studies as is the lack of standardization for pre-operative assessments (including MRI) and criteria for referral for adjuvant treatment.

Patients in the LACC study included women from thirteen countries who had cervical cancer stages ranging from 1Ai (LVSI) to 1Bi. A total of 51 from 631 women (8.1%) had stage 1aii disease and under. Guidelines from ESGO/ESTRO/ESP (Cibula et al., 2018) state that a parametrial dissection is not necessary for stage 1aii disease and under and the study probably does not completely reflect practice in Europe. At the other end of the spectrum, it is apparent that those cases with recurrence often had large tumours (>2cm) and other poor prognostic features such as grade 3 disease, deep stromal invasion, and lympho-vascular invasion. Patients with multiple histological risk factors for recurrence at diagnosis who would require adjuvant therapy should be offered chemo-radiotherapy without previous radical pelvic surgery according to European guidelines (Cibula et al.,2018). In the LACC study, most of the recurrences occurred in patients with large tumours. A supplementary table shows the histological characteristics of the patients who recurred in each arm but regrettably, tumour size and invasion are not included. If this had been included in this table, the reader would have been able to see which women would not have been offered surgery in their own institution. However, 27.6% and 28.8% of patients required adjuvant therapy in the open and MIS arms respectively. The proportion of women requiring adjuvant therapy was higher with exclusion of the cases with stage 1a disease.

There are a lot of positive aspects to this study and completing a RCT for cervical cancer surgery is a huge achievement in its own right. One concern is the early closure, with an enrolment of 85% of the planned participants. The published protocol includes a quality of life aspect of the study and we hope that the authors will publish this data in due course.

## Discussion

The two studies discussed above have changed the evidence balance from one where MIS radical hysterectomy for cervical cancer was thought to have less complications with an equivalent survival to one where it is uncertain if the survival following MIS is as good as open surgery and if the complication rates are any better (Shazly et al., 2015; Sert et al., 2011; Shah et al., 2017; Corrado et al., 2018; Gortchev et al., 2012; Wright et al., 2012; Cantrell et al., 2010; Estape et al., 2009; Frumovitz et al., 2007; Holloway et al., 2007; Ind et al., 2018; Ko et al., 2008; Lowe et al., 2009; Maggioni et al., 2009; Matanes et al., 2018; Nam et al., 2010; Sert et al., 2016; Soliman et al., 2013; Martin-Hirsch et al., 2019). However, more recent studies have shown that MIS seems to be safe, with decreased morbidity and costs in women with tumours of less than 2cm but not in women with larger tumours (Corrado et al. 2018; Kim et al., 2019; Hwang et al., 2012; Margul et al., 2018).

The reasons for the results of the two studies have to be considered. It is possible that the results may be biased by factors already discussed. However, it seems logical that every measure that could be taken should be in case the hypothesis is true. One option would be to abandon all MIS for surgery in cervical cancer. However, if the complication rates for MIS are less, then that policy could also cause harm to patients. If MIS for cervical cancer results in more recurrences than open surgery, then we have to consider why.

The first reason why MIS might cause more recurrences that open surgery in cervical cancer is due to case selection. This is unlikely to have altered the results of a RCT but could account for the results in the Melamed study (Melamed et al., 2018). A management plan should be made only after careful consideration of all aspects of a case with a multidisciplinary team approach, and appropriate counselling with the patient. This area and other aspects of care for cervical cancer is covered in the ESGO/ESTRO/ESP guideline on cervical cancer (Cibula et al., 2018).

Another reason for the potential findings in the two studies might be related to a less radical approach to surgery in the MIS arm. This might be a competency-based issue. Therefore, it seems intuitive to recommend that experienced surgeons are present during such procedures and that such operations are only undertaken in centres of high volume. The guidance falls short of specifying the numbers required as this is unknown. However, with evidence that the number of radical hysterectomy procedures is dramatically falling, (Shah et al., 2017) and that from the literature data an acceptable level of surgical proficiency in LRH is from at least 25 up to 50 cases (Hwang et al., 2012; Kong et al., 2015), it is sensible that institutions should keep a careful audit of their outcomes both in terms of survival, complications and quality of life.

A number of other factors have been proposed as reasons for a possible lower survival rate in MIS compared to an open approach for cervical cancer. These include the use of uterine manipulators, the carbon dioxide (CO_2_) pneumoperitoneum (Mo et al., 2014; Binda et al., 2014) and tumour contamination at colpotomy. Although the effects of these are unknown and the usage was not reported in either of the two new studies (Melamed et al., 2018; Ramirez et al., 2018) it is instinctive that a surgeon should try to minimise any theoretical risk from these sources. Methods proposed in the past have included low CO_2_ pressures, washing with ringer solution, tying off the Fallopian tubes, not using a manipulator, sewing the vagina from below to seal off the cervical tumour, removing the bulky part of the tumour at the beginning of the operation, stapling the vagina and bringing the cervix and tumour into a vaginal tube with the assistance of a suture. Furthermore, it is sensible that lymph nodes should be placed in bags when stored in the abdominal cavity and for retrieval.

Recent preliminary results from the retrospective European SOCCOR study underlined that the use of a manipulator worsened the outcome among MIS patients (Chiva et al., 2019). Moreover, Kohler et al. underlined how a combined laparoscopic-vaginal technique for radical hysterectomy with the avoidance of spillage and manipulation of tumour cells provides excellent oncological outcomes for patients with early cervical cancer (Kohler et al., 2019)

The next challenges will be to standardise the surgical steps to perform MIS radical hysterectomy and to undertake further RCTs to define the correct indications for MIS approaches. The use of video technology has advanced substantially since the onset of these studies and future research should consider including video evidence of every procedure.

ESGE and SERGS released a joint statement in July 2019 to highlight this debate and outline the opinion of the two societies on this debate (Appendix).

## Conclusion

The joint statement presented here is based on two new studies and pre-existing evidence. It is inevitable that new evidence will emerge over the next few years that will supersede that presented in this document and will result in modification of existing guidelines. For now, we do not recommend the abandonment of MIS for cervical cancer. For tumours of > 2cm, MIS should be considered predominantly within clinical trials. However, the two papers described in this document will inevitably make surgeons reflect on how they counsel their patients and how they manage their surgery intra-operatively.

## Appendix

Joint Statement by the European Society for Gynaecological Endoscopy (ESGE) and the Society of European Robotic Gynaecological Surgery (SERGS) https://esge.org/wp-content/uploads/2019/08/ESGE-SERGS-Statement.pdf

Common Working Group in Oncology ESGE: Mereu Liliana, Rovira Negre Ramon, Habib Nassir, Scambia Giovanni

SERGS: Ind Thomas, Kimming Rainer, Zanagnolo Vanna, Verheijen Rene HM

July 2019

Approved by the Executive Boards of ESGE & SERGS after open review in the websites of both societies

- The surgical management of cervical cancer is described in detail in a guideline from European Society of Gynaecological Oncology (ESGO) / European Society for Radiotherapy and Oncology (ESTRO) /European Society of Pathology (ESP) (Cibula et al.,2018) The general recommendations for the management of cervical cancer are that:
- Treatment planning should be made on a multidisciplinary basis (generally at a tumour board meeting) and based on prognostic factors for oncological outcome, morbidity and quality of life.
- Patients should be carefully counselled about the suggested treatment plan as well as potential alternatives. This should include the risks and benefits of all the available options.
- Treatment should be undertaken by a team of specialists dedicated to the diagnosis and management of gynaecological cancers.
- The lead surgeon for a radical hysterectomy for a cervical cancer procedure should be someone who participates in such procedures regularly and has a wealth of experience.
- Centres who perform radical hysterectomy for cervical cancer should audit their outcomes.
- A minimally invasive approach to radical hysterectomy either by standard laparoscopy or robotics can still be considered.
- When considering a Minimally Invasive Surgery (MIS) radical hysterectomy for cervical cancer, women should be informed of all the evidence concerning the route of surgery in terms of complications and survival. The present evidence is;
- Many observational studies have shown no differences in survival between MIS and open surgical approaches.
- Two recent studies, the randomised study by Ramirez et al. and the epidemiologic study by Melamed et al. found that MIS radical hysterectomy for cervical cancer was associated with shorter overall survival than open surgery.
- All the studies were unable to find a difference in survival between MIS and an open surgical approach in the subgroup of women with tumours <=2 cm.
- The randomised study by Ramirez et al. has shown a significantly better survival using open surgery for cervical cancer for large tumours (>2cm).
- Both recent studies were unable to explain why MIS was associated with shorter survival.
- Many observational studies have shown a reduced complication rate for MIS compared to an open surgical approach.
- A recent randomised controlled study showed no difference in complication rates between an open and minimally invasive approach.
- During a radical hysterectomy, every effort should be made to avoid tumour cell spillage and contamination of the peritoneum during surgery. Techniques that have been employed include sewing closed the vagina prior to disconnection of the uterus, using a vaginal stapling device, and bringing the cervix into a vaginal tube using a suture. Furthermore, areas for consideration include techniques such as reducing unnecessary uterine manipulation; avoiding excessive intra-abdominal carbon dioxide pressures; and placing lymph nodes in bags rather than leaving them free in the pelvic peritoneum.
- ESGE and SERGS support the concept of confirmatory controlled trials.
- ESGE and SERGS support the concept of a standardised methodology for MIS radical hysterectomy for cervical cancer.
